# Integrated transcriptional analysis reveals macrophage heterogeneity and macrophage-tumor cell interactions in the progression of pancreatic ductal adenocarcinoma

**DOI:** 10.1186/s12885-023-10675-y

**Published:** 2023-03-02

**Authors:** Kaidi Yang, Tongxin Yang, Jian Yu, Fang Li, Xiang Zhao

**Affiliations:** 1 Department of Oncology, Hainan Hospital of Chinese People’s Liberation Army General Hospital, Sanya, 57200 Hainan Province P.R. China; 2 Department of Oncology, Southwest Hospital, Third Military Medical University (Army Medical University), Chongqing, P. R. China; 3 Department of Health Statistics, Naval Medical University, Shanghai, 200433 PR China

**Keywords:** Single-cell RNA sequencing, Pancreatic ductal adenocarcinoma, Prognosis, Macrophage heterogeneity, Tumor-macrophage interactions

## Abstract

**Background:**

Pancreatic ductal adenocarcinoma (PDAC) is a highly lethal disease harboring significant microenvironment heterogeneity, especially for the macrophages. Tumor-associated macrophages (TAMs) orchestrate PDAC malignancy, but their dynamics during disease progression remains poorly understood. There is a pressing need to identify the molecular mechanism underlying tumor-macrophage interactions and thus design novel therapeutic strategies.

**Methods:**

Herein, we developed an insilico computational method incorporating bulk and single-cell transcriptome profiling to characterize macrophage heterogeneity. *CellPhoneDB* algorithm was applied to infer macrophage-tumor interaction networks, whereas pseudotime trajectory for dissecting cell evolution and dynamics.

**Results:**

We demonstrated myeloid compartment was an interactive hub of tumor microenvironment (TME) essential for PDAC progression. Dimensionality reduction classified seven clusters within the myeloid cells wherein five subsets of macrophages were characterized by diverse cell states and functionality. Remarkably, tissue-resident macrophages and inflammatory monocyte were identified as potential sources of TAMs. Further, we uncovered several ligand-receptor pairs lining tumor cells and macrophages. Among them, *HBEGF-CD44*, *HBEGF-EGFR*,* LGALS9-CD44*, *LGALS9-MET*, and *GRN-EGFR* were correlated with worse overall survival. Notably, as in vitro experiments indicated, TAM-derived HBEGF promoted proliferation and invasion of the pancreatic cancer cell line.

**Conclusion:**

Together, our work deciphered a comprehensive single-cell atlas of the macrophage compartment of PDAC and provided novel macrophage-tumor interaction features with potential value in developing targeted immunotherapies and molecular diagnostics for predicting patient outcome.

**Supplementary Information:**

The online version contains supplementary material available at 10.1186/s12885-023-10675-y.

## Introduction

Pancreatic ductal adenocarcinoma (PDAC) is one of the most lethal cancers with a 5-year survival rate of less than 8% [1]. The intractable characteristics of PDAC are the result of joint factors like population demographics, genetics, personal health status, immunity condition, and environment, which relate to the sequential accumulation of driven mutations (*KRAS*, *CDKN2A*, *TP53*) and passenger mutations during tumor progression, and confers tumors with marked cellular and molecular heterogenicity [2–4]. PDACs develop in a complex tumor microenvironment (TME) that dynamically interacts with tumor cells to assist tumor growth and progression [5]. Among the diverse cellular components in TME, tumor-associated macrophages (TAMs) appear early during pancreatic tumorigenesis and represent the predominant stromal cell fractions that elicit severe T-cell exhaustion and eventually overwhelm antitumor cellular immunity in the advanced lesion [6, 7]. It is noteworthy that TAMs contribute to nearly all aspects of cancer progression, including angiogenesis, anti-inflammation, extracellular matrix (ECM) remodeling, and raising treatment resistance upon gemcitabine administration [8–10]. Therefore, understanding the molecular mechanisms governing tumor-macrophage interactions is critical. Recent advances in computational biology and muti-omics technologies enabled deciphering cell-cell communication from high-throughput transcriptomic data [11]. By profiling molecular spectrums of intracellular tumor-macrophage communication, we are able to design targeted molecular therapies to tackle PDAC.

Optimal therapeutic strategies targeting TAMs require an in-depth understanding of their origins, fates, and dynamics. Current advances in single-cell technology enable the characterization of cell trajectory inference and phenotypic evolution of macrophage compartments [12], while it remains poorly characterized for PDAC. It is a traditional view that TAMs are derived from the recruited monocytes [13]. However, emerging evidence suggests resident tissue macrophages (RTMs) of embryonic origin as the sources of TAMs. These cells exhibit unique pro-fibrotic functions distinct from monocyte-derived counterparts [14]. RTMs are established during the embryonic development stage and maintained by self-renewal locally in addition to recruitment [15]. Since tissue-specific niche has a major influence on environmental reprogramming of seeded embryonic precursors, RTMs that originated from different organs or tissues are conferred with distinct phenotypes and functions [16, 17]. It was found that RTMs of the pancreas expanded during tumorigenesis and contributed to cancer progression [14], while their phenotypic characteristic and lineage relationship with TAMs, especially at a single-cell level, remains to be elucidated.

According to the induced conditions and activation states, TAMs are classified into M0-type (naive macrophage), M1-type (classically activated macrophage), and M2-type (alternatively activated macrophage) [18]. Researchers were used to this typing system for a long time, while recent studies on the genetically engineered mouse model of PDACs, as well as human primary gastric cancer, revealed no such divisions with single-cell technology [19, 20]. M1/M2 division system was based on in vitro artificial induction and thus might not be applicable for the real in vivo environment. Instead, applying single-cell RNA sequencing (scRNA-seq) to the comprehensive dissections of pan-cancer macrophage ontogeny has revealed distinct features of tumor-infiltrating macrophages across cancer types [12]. Further study on colon cancer has revealed dichotomous subtypes of TAMs (C1QC + and SPP1 + TAMs), which demonstrate unique transcriptional profiles [21]. Intriguingly, these two functional TAM subsets exhibited enrichment of inflammatory and angiogenic signatures respectively, emphasizing the importance of classifying macrophages according to functional diversity.

Herein, we performed computational analyses to delineate the features of diverse macrophage subsets and construct novel macrophage-tumor cell interaction networks involved in regulating the malignancy of PDACs. Differentiation trajectories analysis showed that RTMs and inflammatory monocytes are potential sources of TAMs. Our study uncovered cellular reprogramming programs of the macrophage compartment and provided a strong rationale for personalized/precision approaches for macrophage-directed cancer therapy.

## Materials and methods

### Generation of the primary PDAC dataset

The bulk gene expression and survival data (n = 182) of TCGA pancreatic cancer were downloaded from the UCSC data portal ( https://xenabrowser.net ). The raw count data obtained from the RNA-sequencing (RNA-seq) was normalized concerning library size using the Deseq2 package, thus making fair gene comparisons between samples. After excluding cases not histologically diagnosed as primary ductal cancer and deficient in survival data, we reserved 147 PDAC patients for further analysis.

### Single-cell data processing

The published PDAC single-cell dataset [22] containing 24 tumors and 11 normal pancreas tissues was deposited in the Genome Sequence Archive (https://ngdc.cncb.ac.cn/gsa/) with the accession number PRJCA001063. We retrieved the raw data matrix and transformed it into a *SingleCellExperiment* object using the *Seurat* R package. After removing cells with poor quality (< 200 genes/cell, < 10 cells/ gene, and > 10% mitochondrial genes), the raw matrix was processed using “*Sctransform*” methods, which contains encapsulated functions of “*NomalizeData*”, “*ScaleData*”, and “*FindVariableFeatures*” for automatically identifying high-variance gene. Next, the ElbowPlot was generated for determining the optimal dimensionality number of principal components (PCs). As high resolution often leads to an increasing number of clusters, the “*FindClusters*” function for applying modularity optimization was adjusted to 0.15. The UMAP (Uniform manifold approximation and projection), a dimension reduction technique, was used for visualizing the clustering. To assign cell identity to each cluster, previously published literature and *CellMarker* dataset (http://biocc.hrbmu.edu.cn/CellMarker) were used for reference. Following identifying the myeloid cells/macrophages subset according to expressions of *AIF1* and *CD68*, the same workflow was used for making further sub-classification. To perform over-representation analysis (ORA), we identified differentially expressed features (DEGs) for each subset against other subsets using the “*FindMarkers*” function in which parameters of min.pct (minimum percent of cells expressing the gene) and log2 fold-change are set to 10% and 0.5, respectively. Pathway enrichment analysis was performed using the *ClusterProfiler* package.

### Correlative cell-cell interactions inferred by combined bulk and single-cell transcriptome profiling

To conduct correlative cell-cell interaction analysis, we refer to the previously published protocol [21]. Based on the TCGA bulk profiling, we estimated the relative abundance of each cell type by the average expression of the cell type-specific genes defined in the single-cell data processing. Subsequently, Pearson Correlation analysis was performed between each gene and the relative abundance of each cell type to identify genes that may infer the co-occurring cells with these particular cell types. Since the expression levels of specific signature/marker genes in a given cell subtype (*i*) were obviously in high correlation with the abundance of this specific cell subtype, we filtered these genes according to the criteria of average expression > 1 and cell frequency of expression > 20% upon scRNA-seq dataset. Next, the top 20 highly correlated non-self-expressed genes for cell-type *i* were selected based on the ranked correlation coefficient matrix. To identify certain cell types in relation to cell-type *i*, we searched the candidates contributing to the highly correlated non-self-expressed genes by performing gene set enrichment analyses through calculating the mean expressions of these selected genes across all cell types followed by the application of z-score transformation. At last, the correlated cell types for cell-type *i* were identified if the z-score transformed enrichment score over 1.28. If the correlated two types were identified mutually, the maximum of the enrichment scores was chosen.

### Calculate module scores for feature expression programs in single cells

To delineate the functional state/signature for each macrophage sub-cluster, the *AddmoduleScore* function integrated into the *Seurat* package was used for computing the module scores of annotated gene sets from the *MSigDB* hallmark collection (https://www.gsea-msigdb.org/gsea/msigdb/). Then, we performed differential expression gene (DEG) analysis using the *Seurat::FindAllMarkers* function with a log2 fold-change threshold set to 0.5 and *P*-value < 0.01 to choose the top 2 or 3 pathways across macrophage clusters.

### Single-cell regulatory network inference and clustering (SCENIC)

For single-cell regulons (i.e., transcription factors and their target genes) inference in the different subsets of macrophages, the SCENIC protocol (https://aertslab.org/) was followed for reference [23]. The inference of regulons was performed in the following three steps. (1) Co-expression modules between transcription factors and their potential target were identified based on the gene-expression matrix through *GENIE3* (R package) (2) To remove indirect targets, modules were pruned by cis-regulatory motif discovery (cisTarget), leaving 94 regulons analyzed in the next step. (3) The activity score of each regulon at cellular resolution was computed using the *AUcell* algorithm and subsequently used for t-SNE dimensionality reduction and visualization.

### Cell-cell communication inference

To systematically study the interactions between macrophage and cancer cells, we predicted cell-cell communication via *CellphoneDB* (https://www.cellphonedb.org/), a publicly available repository of ligand-receptor interacting pairs and multi-subunit protein complex [24]. It considered the expression level of ligands and receptors with each cell type, generated a null distribution of each ligand-receptor pair, and determined the likelihood of cell-specificity of a given receptor-ligand complex based on the *P*-value. Those with the least number of significant *P*-values are chosen to be biologically relevant. After filtering in a criterion of *P* < 0.05 and containing secreted factors, a total of 92 ligand-receptor pairs were identified, as shown in Supplementary Table 1. Special mention should be made that *CellphoneDB* generates significant interactions between specific cell pairs in two forms: *Macrophage-Tumor pair* and *Macrophage-Tumor pair*, for which different ligand-receptor pairs are enriched.

### Trajectory analysis

The *monocle2* package was used to build the developmental trajectory of macrophages. Following the protocol, normalized data of the macrophage subset served as input to establish the *monocle* object. Next, the top 2,000 most significant (q < 0.1) DEGs were taken as the set of ordering genes and sorted by the q-value. To isolate the root-to-branch specific gene expression patterns and trajectory modeling, the monocle *BEAM* function was used to compare two models with a likelihood ratio test for branch-dependent expression. For visualizing the fate-dependent gene expression patterns and expression dynamics for each gene, “*plot genes branched heatmap*” and “*plot genes branched pseudotim*e” functions were used.

### Cell culture and differentiation

THP-1 cell line (human monocytic leukemia cell line) and SW1990 (human pancreatic cancer cell line) were purchased from American Type Culture Collection (Manassas, VA). Cells were cultured in Dulbecco’s modified Eagle’s medium (DMEM) supplemented with 10% fetal calf serum, penicillin, and streptomycin in the presence of 5% CO2. THP-1 cells were induced to be polarized into an attached macrophage-like phenotype by stimulation with 150 nM PMA (Sigma-Aldrich, Shanghai, China) for 24 h. Subsequently, M2 differentiation can be achieved by the combined treatment with 25 ng/ml IL-4 and 25 ng/ml IL-13. To build a co-culture system, 1 × 10^5^ cells of polarized macrophages and SW1990 cells were co‑cultured with a Transwell apparatus of 0.4 μm pore hanging inserts (Corning, USA) for three days. Neutralization handling was performed using HBEGF Polyclonal Antibody (PA5-47352, Invitrogen, USA).

### Quantitative reverse transcription PCR (RT-qPCR)

According to the manufacturer’s protocol, the total RNA was extracted using RNA fast 2000 Reagent (Fastagen, Shanghai, China). Purified RNA was reverse‑transcribed using oligo-dT and random primers with the PrimeScript™ RT reagent Kit (Takara, Dalian, China). RT‑qPCR was run on a CFX-96 real-time PCR System (Bio-Rad, Shanghai, China) using TB GreenTM Premix Ex Taq II (Takara, Dalian, China). The sequences of the primer sets for *GRN*, *LGALS9*, *HBEGF*, and *GAPDH* were listed in Supplementary Table 2. The relative expression for each gene was normalized to GADPH and calculated using the 2^−ΔΔCT^ formula.

### Invasion assay

Transwell assay was performed to assess cell invasion using 6-well Matrigel pre-coated cell culture inserts (Corning, USA). 1ml aliquot (4 × 10^5^) of preprocessed SW1990 cells in DMEM medium (1% FBS) was plated into the upper chamber of each insert and filled the lower chamber with 2 ml complete medium. After 24 h, non-invading cells were removed from the upper surface using cotton swabs, whereas invaded cells on the bottom layer of the surface were rinsed in 1×PBS, fixed with pre-chilled 4% paraformaldehyde solution for 30 min, stained with 0.1% crystal violet dye for 10 min and followed by rinsing in 1×PBS for three times. For imaging acquisition, dried inserts were observed under an inverted light microscope.

### Cell proliferation assay

Briefly, 1 × 10^3^ preprocessed SW1990 cells were seeded in a 96-well plate. The medium was replaced with 100 µl fresh medium with 10% Cell Counting Kit-8 (CCK-8) reagents (Beyotime Biotech, China) in each well at 0, 24, 48, and 72 h and incubated at 37 °C for additional two hours. Finally, the absorbance at 450 nm was measured using an ELISA microplate reader (Thermo, USA).

### Statistical analysis

For cell-cell communication inference, the means of the average expression level of interacting molecule1 in malignant ductal cells and interacting molecule2 in macrophages are calculated using the square root of mean((molecule1 × molecule2) + 1). The Kaplan-Meier survival curve was built to show differential survival between two groups, and a log-rank statistical test was performed to compare the survival distributions of the two groups (*P <* 0.05). We defined the “High” and “Low” by setting the cut-off as a median value of the gene expression level or gene signature scores. To compare non-normal distribution parameters between two groups, unpaired two-tailed Wilcoxon rank-sum tests were performed. By contrast, normally distributed data were compared between the two groups using the Student’s t-test. All statistical analyses were performed using Graphpad Prism 8.0 or R version 3.6.3.

## Results

### Identifying myeloid cell subset as an interactive TME hub essential for PDAC progression

To interrogate the global cell interaction atlas of PDACs, we incorporated scRNA-seq and TCGA bulk transcriptome datasets into the integrated analysis. From the published scRNA-seq profiles, a total of 10 cell subsets were identified and annotated as previously defined [22]. Gene signatures detected in over 25% of each cell population with a log2 fold-change greater than 0.5 were identified (Supplementary Table 3). By identifying the abundance of a particular cluster in each TCGA sample using its gene signatures, we generated non-self highly correlated genes and matched this gene set back to scRNA-seq dataset. Co-occurring enriched cell types were identified and used for building cell-cell interaction networks (Supplementary Fig. 1a). It was found that myeloid cells and malignant ductal cells harbor the greatest number of partners, suggesting their close relationships with TME components (Supplementary Fig. 1a). Increased myeloid cell infiltration informed a worse prognosis for PDAC patients (Supplementary Fig. 1b). Thus, we termed myeloid cell compartment as an interactive hub essential for cancer progression. In contrast, there were no similar interactions in normal pancreas tissue. Functional enrichment analysis of the DEGs between tumor and normal myeloid cells (Supplementary Fig. 1c) revealed that digestion- and metabolism-related functional modules were enriched in normal tissue, whereas genes up-regulated in tumor samples were significantly enriched for immune-associated terms, such as interferon signaling, interleukin 4 and interleukin 13 signalings, and toll-like receptor cascades (Supplementary Fig. 1d).

### Identification of macrophage heterogeneity

**Fig. 1:**
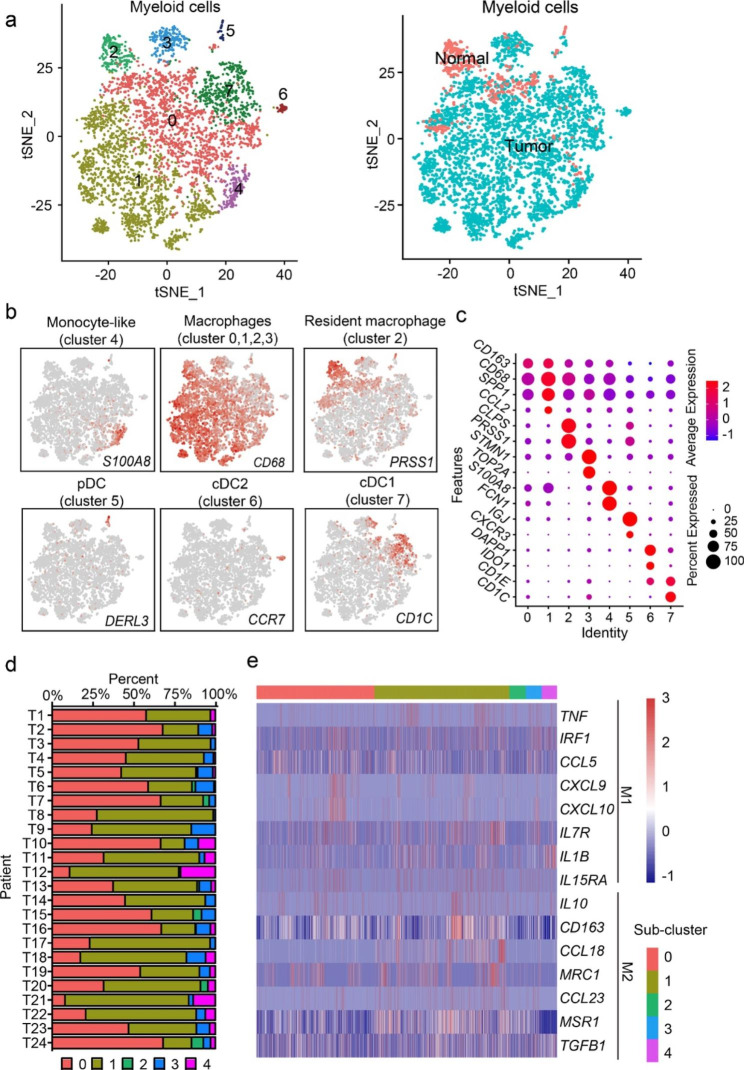
**Identification of macrophage heterogeneity. a** t-SNE projection of 5408 myeloid cells from PDAC tumors and normal tissues, colored according to graph-based clusterings (left panel) or sample origin (right panel). **b** t-SNE plots showing normalized expression of *S100A8, CD68, PRSS1, DERL3, CCR7, and CD1C*, representative cell markers for specific cell types. **c** Dot plot depicting expression levels of representative marker genes across different myeloid subsets. **d** Hundred-percent bar chart illustrating the distributions of different macrophage subsets across PDAC samples. **e** Heatmap illustrating expressions of M1 and M2 markers across different macrophage subsets. pDC, plasmacytoid dendritic cell; cDC, conventional dendritic cell; t-SNE, t-distributed stochastic neighbor embedding

To profile myeloid cell diversity, the coarse-grained annotated myeloid cluster from tumor and normal tissue were sub-divided into eight subsets (Fig. 1a). Of note, DC subsets were characterized by high expression of *HLA-DRs* and low expression of *CD68*, and further distinguished by specific expression of *DERL3/CXCR3/IGJ*, *CCR7/IDO1*/*DAPP1*, and *CD1C*/*CD1E* for plasmacytoid DC (pDC), cDC2, and cDC1 cells, respectively (Fig. 1b-c). The remaining five clusters were denoted as macrophages concerning the high expression of *CD68* and *CD163* (Fig. 1b-c). Intriguingly, no prominent batch effect was observed across the patients, and macrophage subsets were shared with diversity among patients, albeit at different proportions, showing intertumoral heterogeneity (Fig. 1d and Supplementary Fig. 2a). Gene signatures for each subset are identified and presented in Supplementary Table 4. Among the diverse macrophage subsets, cluster 2 macrophage showed preferential enrichment (91.9%) in normal mucosa relative to the tumor and hence, was designated as resident tissue macrophages (RTMs) (Fig. 1a and Supplementary Fig. 2b). In addition to RTMs, cluster 0 macrophage formed the majority of macrophage subset (35.7%) in normal tissue (Supplementary Fig. 2b). Cluster 4 macrophage was assigned into inflammatory monocytes regarding the high expression of *S100A8, S100A9, VCAN, FCN1* [25]. Cluster 0 macrophage showed elevated expression of MHC-II class molecules (*HLA-DPB1, HLA-DPA1, HLA-DQB1*), whereas cluster 1 macrophage displayed higher expression levels of cytokines *CCL2, CCL3, ADM*, et al., apolipoprotein genes *APOC1, APOE*, and pro-fibrotic genes *SPP1, TIMP1* in control of ECM deposition [26]. Cluster 2 macrophage, known as RTM, exhibited specific signatures expression (*PRSS1, CLPS, SYCN*). The immunohistochemical analysis confirmed the over-expression of these newly defined markers in normal pancreas using the online resource (Human Protein Atlas Dataset, Supplementary Fig. 3a-c). Further, cluster 3 macrophage was characterized by high expression of cell-cycle genes (*TOP2A, CDK1*, *MKI67*) and thus termed as cycling cells (Fig. 1c and Supplementary Fig. 2c). Deep analysis of M1 (e.g. *CXCL9, IL1β, CCL5*) and M2 (e.g. *MRC1, CCL18, CCL23, CD163*) signatures across the subsets indicated subpopulation of cluster 1 macrophage highly expressed central regulators (*IRF1*, *CXCL9*, and *CXCL10)* in Type-I interferon (IFN-I) signaling, resembling previously defined “ISG” macrophage that exhibited an M1 phenotype bias induced by IFN-I [12, 27]. Even so, there was no recognizable subset representative of M1- or M2- polarized macrophage (Fig. 1e).

### Delineation of the functional states across different macrophage subsets

**Fig. 2:**
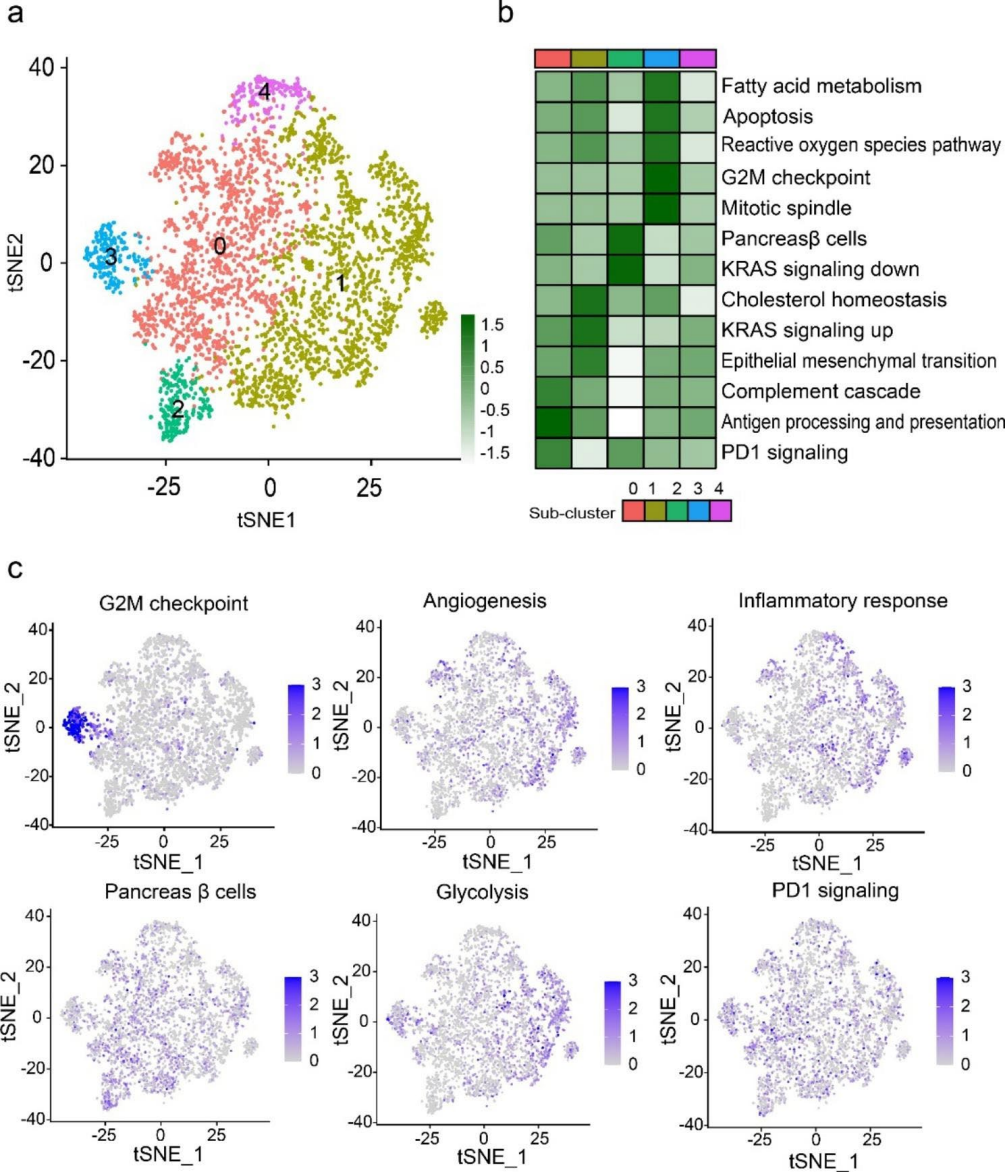
**Delineation of the functional states across different macrophage subsets**. **a** t-SNE projection of cluster 0–4 macrophages, colored according to cell identity. **b** Heatmap showing the normalized single sample gene set enrichment analysis (ssGSEA) score for cancer- and immunological-related gene sets from the *MSigDB* hallmark collection across different macrophage subsets. **c** t-SNE projection of normalized ssGSEA score for representative hallmark and immune terms.

To delineate the functional states across macrophage subsets, we performed singlesample gene set enrichment analysis (ssGSEA) and over-representation analysis (ORA) in terms of immune- and cancer-related phenotypes, which aggregate redundant biological states or processes. Our data indicated that cluster 0 macrophage displayed upregulation of pathways associated with complement cascade, antigen processing and presentation, and PD1 signaling, whereas cluster 1 macrophage was characterized by active metabolism and stroma, relating to properties of TAMs (Fig. 2a-c and Supplementary Fig. 4). Consistent with above finding, cluster 3 macrophage exhibited preferential enrichments of cell-cycle related terms (Fig. 2a-c and Supplementary Fig. 4). Processes of inflammation and innate immune response, indicative of monocyte-like properties, were enriched in cluster 4 macrophage (Supplementary Fig. 4).

### Identification of master regulons across macrophage subsets

**Fig. 3:**
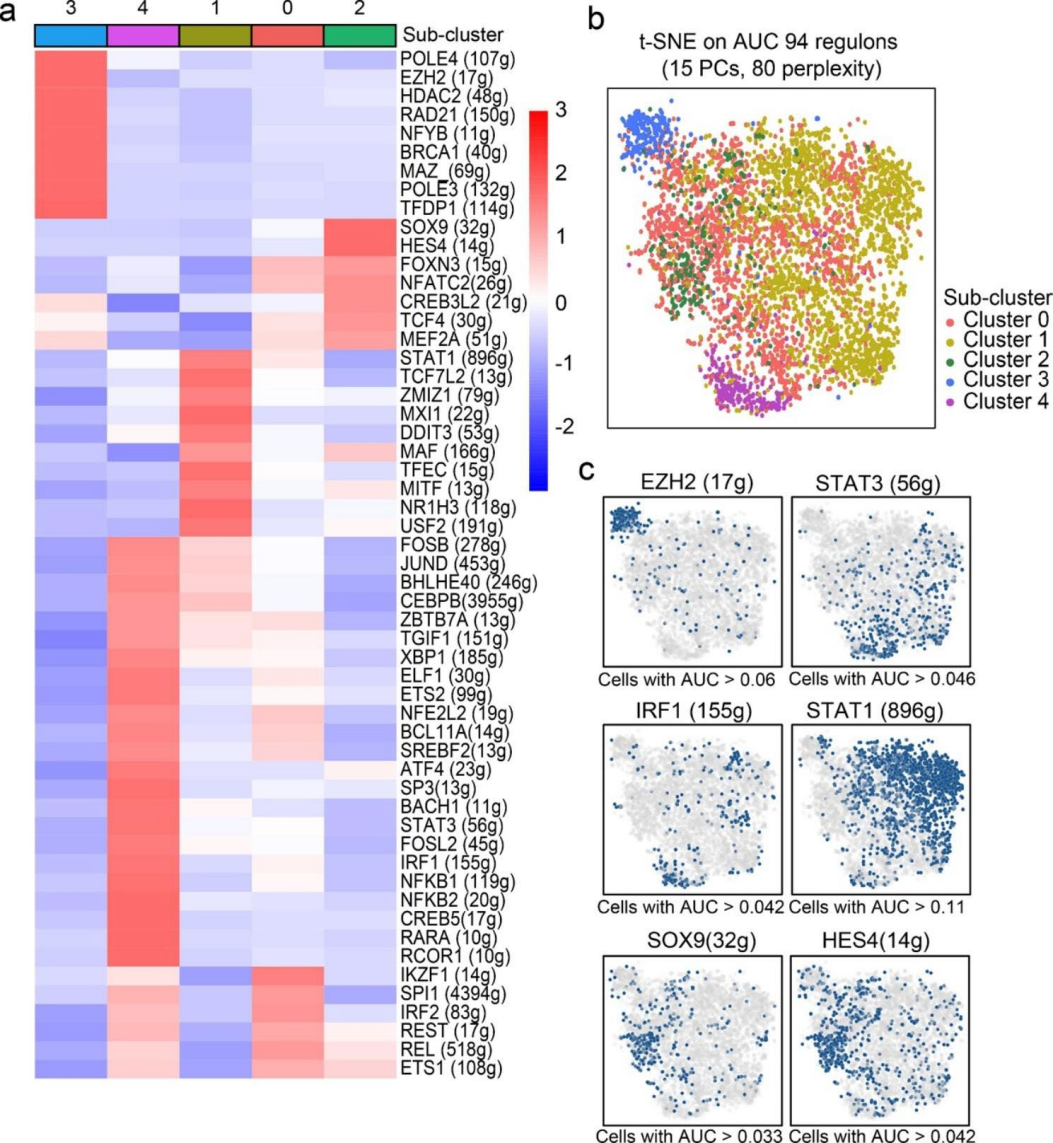
**Identification of master regulatory networks across macrophage subsets. a** Heatmap showing the normalized activity score of representative regulons across different macrophage subsets. **b** t-SNE plots based on activity score of 94 regulons and colored by annotations inferred from whole transcriptome profiling. **c** t-SNE plots showing activities of IRF1, STAT3, EZH2, KLF5, TCF4, and HES4 regulons. *g included in the parenthesis represents the number of genes in the regulons. PC, principal component; AUC, the area under the curve

Building transcriptome-based regulatory networks, also known as regulons, contributes to a better understanding of immune cell function and regulation [28]. We generated macrophage subset-specific core regulons for determining and characterizing cellular state using SCENIC (Fig. 3a). It was observed that clustering by cell state was compatible with the division using whole transcriptome profiles (Fig. 3b), showing good performance for SCENIC. With the analyses, the cluster 4 subset was characterized by the upregulated activity of inflammatory regulons overexpressed in previously defined monocyte [27, 29], including IRF1, CEBPB, STAT3, and NFKB2 (Fig. 3a,c), confirming its classification as an inflammatory monocyte. EZH2, best known for its function in macrophage activation [30, 31], was identified as a candidate regulon driving the rapid-cycling status for cluster 3 macrophage. Additionally, we noted a trend of increased STAT1 activity in cluster 1 macrophage, whereas upregulation of SOX9 and HES4 seemed responsible for the distinct phenotype of RTMs (cluster 2) (Fig. 3c).

### Pseudotime trajectory reconstruction of macrophage reprogramming course

**Fig. 4:**
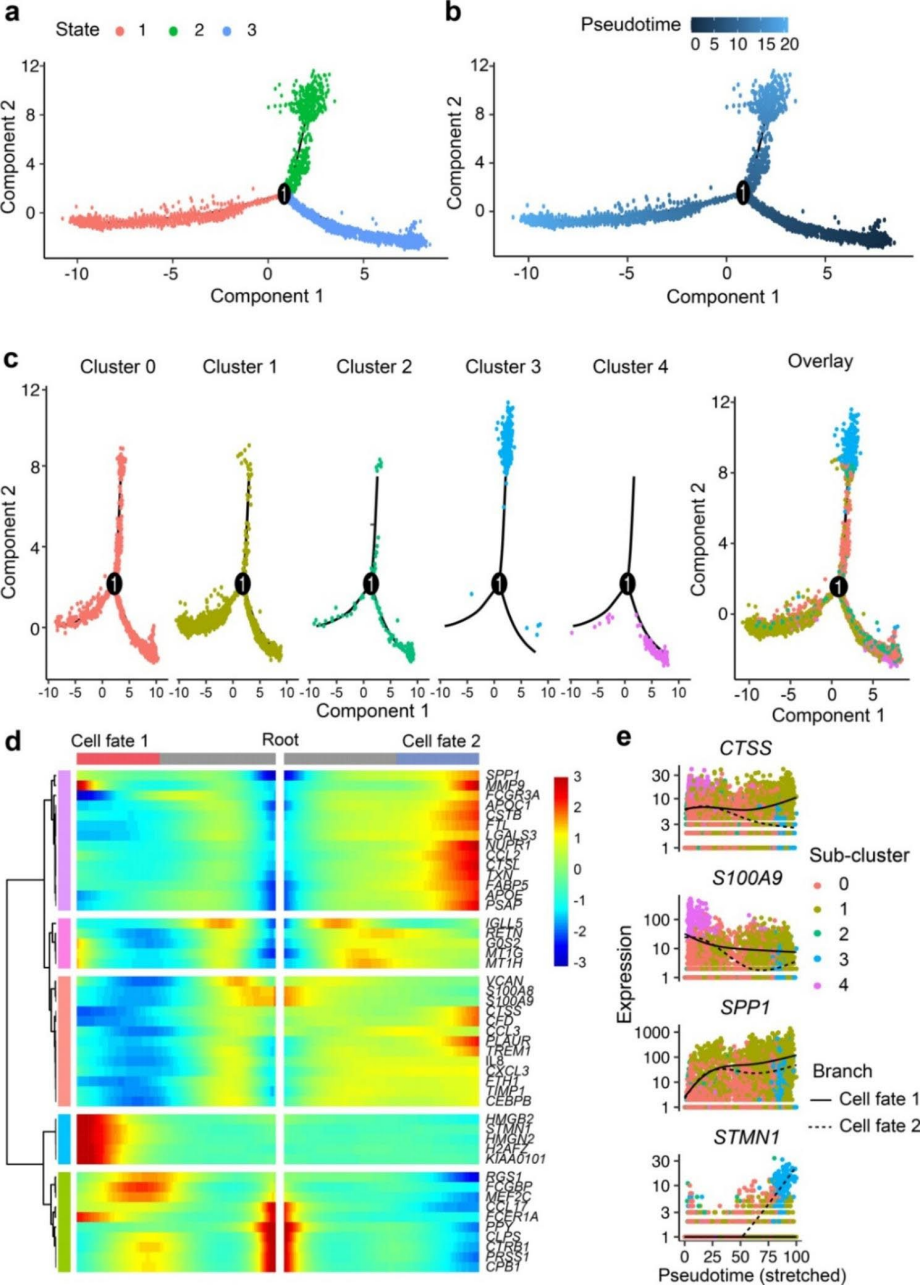
**Pseudotime trajectory reconstruction of macrophage reprogramming course. a,b** Pseudotime trajectory was reconstructed on the whole macrophage compartment containing one branch point. Cells of different States are denoted with different colors (left panel). Cells are highlighted according to the pseudotime ranging from 0 to 20 (right panel). **c** Each macrophage subset (left panel) and whole macrophage subsets (right panel) were projected onto the trajectory plots with different colors assignment. **d** Heatmap presents a differentially expressed genes by comparing two cell states (q < 10^− 50^), each row represents expression level of each gene along the branch trajectory. **e** Pseudotime kinetics of representative genes from the root of the trajectory to cell fate 1 (solid line) or cell fate 2 (dashed line), with each dot representing a single cell and color-coded by cell identity

Pseudotime trajectories provide novel insights for understanding transcriptomic dynamics and discovering developmental regulation mechanism. With the pseudotime analysis, the macrophage subset was ordered along the trajectory comprising one starting point (root) and two termini corresponding to two distinct cell fates (Fig. 4a-b). By projecting the cell identities onto the trajectory, cluster 4 macrophages primarily occupied the root side of the trajectory, confirming that peripheral monocytes are the primary source of infiltrated macrophages (Fig. 4c). Notably, cluster 2 macrophages also served as the progenitor of tumor-infiltrating macrophages, and cluster 3 macrophages were distributed broadly in the termini of State2. Further, State1 and State2 vectors appear to represent differentiation routes for fractions of cluster 0 and cluster 1 macrophages, respectively (Fig. 4c). We posit that these two clusters may populate the pool of fully polarized macrophages. Profiling gene regulation dynamics along the root-cell fate trajectories revealed a reduction of known monocyte markers (*S100A9, S100A8*) and upregulation of *MMP9, CCL2, CTSL* et al. (Fig. 4d). Pseudotime kinetics of *SPP1*, known as a TAM marker [21], showed a gradual upregulation from the root to two fates. In contrast, cells with high expression of *S100A9*, an inflammatory monocyte marker, were preferentially distributed at the beginning of both paths (Fig. 4e). Further, *STMN1* expression exhibited a considerable rise from the late stage of fate 2, while *CTSS* showed divergent expressions along the two trajectories (Fig. 4e).

### Inference of tumor-macrophage interactions

To further investigate the molecular mechanism underlying tumor-macrophage interactions, an unbiased ligand-receptor (L-R) interaction analysis was performed between macrophage subsets and malignant ductal cells according to the expression level and specificity of each ligand-receptor pair. From the interaction strength matrix, we noted that cluster 3 and cluster 1 macrophages showed close interactions with pancreatic tumor cells. By contrast, monocyte-like macrophages (cluster 4) showed the weakest strengths of interactions (Supplementary Fig. 5a). Our deep look into the data showed that cluster 1 macrophage highly expresses cytokines and chemokines (*CCL2, CCL3, CXCL3, CCL4, CX3CL1*, *ADM, CCL18*), with potential roles in maintaining TAM phenotype and function (Supplementary Fig. 5c).

**Fig. 5:**
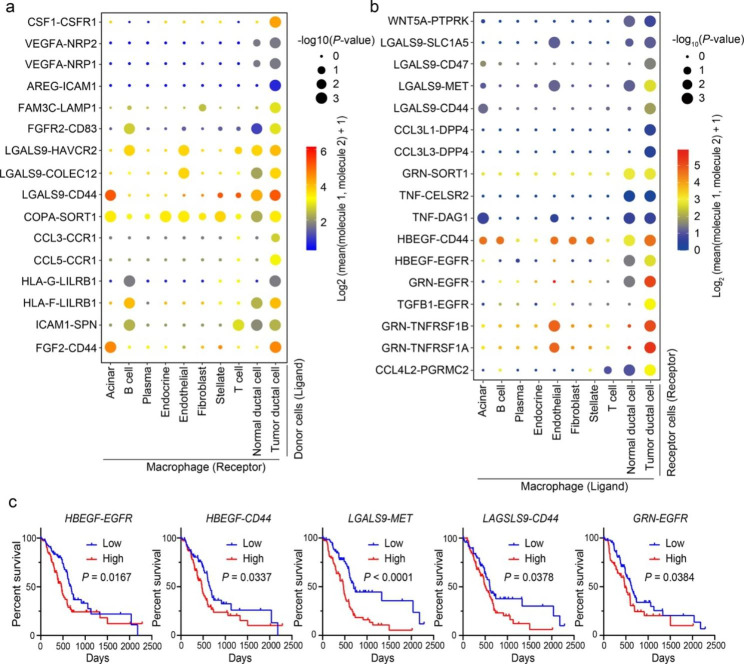
**Inference of tumor-macrophage interactions. a-b** Dot plot illustrating the ligand-receptor pairs with significant meaning for tumor-macrophage interaction across the cell compartments of PDAC. Macrophages were considered as receptor cells (panel **a**) or donor cells (panel **b**) as shown above. *P*-values are indicated by circle size; The means of the average expression level of interacting molecule 1 in cluster 1 and its interacting molecule 2 in cluster 2 are indicated by color. **c** Kaplan-Meier curves of overall survival in different groups of PDAC patients stratified by the median expression level of the ligand and receptor. *P* < 0.05 was identified as significant

Next, we sought to assess the degree of interactions incorporating all the TME components. It is noted that, with regards to macrophages, malignant ductal cells appear to be the closest cooperator (Supplementary Fig. 5b). Master L-R pairs establishing the relationship were presented with forms of Macrophage (Ligand)-Tumor (Receptor) pair (Fig. 5a) and Macrophage (Receptor)-Tumor (Ligand) pair (Fig. 5b). Among them, the *CSF1-CSFR1* axis was present with significant effects on macrophage activation and polarization, whereas *TGFB1-EGFR* axis was reported to mediate tumor migration and invasion [32]. Moreover, this result raised the previous unknown pairs (*CCL3L1-DPP4, LGALS9-MET, TNF-DAG1*), which might serve critical functions (Fig. 5b).

To investigate the value of tumor-macrophage L-R pairs in predicting patient survival. Seventeen L-R pairs with macrophage-source ligands were selected to observe the tumor-promoting effects. Analyses of primary PDAC cohorts from TCGA supported five pairs (*HBEGF-CD44, HBEGF-EGFR, LGALS9-CD44, LGALS9-MET, GRN-EGFR*) as molecular indicators of adverse clinical outcomes (Fig. 5c). Interestingly, *MET* and *EGFR* exhibited a rising trend of expressions in the malignant ductal cells compared with normal ductal cells (Supplementary Fig. 5d). Also, *LGALS9, HBEGF*, and *GRN* were confirmed to be over-expressed in macrophage subset (Supplementary Fig. 5d), highlighting the potential roles in mediating tumor-macrophage interactions. Further, we considered tumor cells as the donor cells to see whether there exist interactions associated with patient survival. Interestingly, the *HLA-F-LILRB1* pair was the only interaction associated with patient survival. *LILRB1* has been reported to exert an immunoregulatory effect via interacting with a wide spectrum of HLA class I molecules [33], thus meditating tumor cell eradication and optimistic prognosis (Supplementary Fig. 5e).

### TAMs-derived HBEGF promotes proliferation and invasion of SW1990

**Fig. 6:**
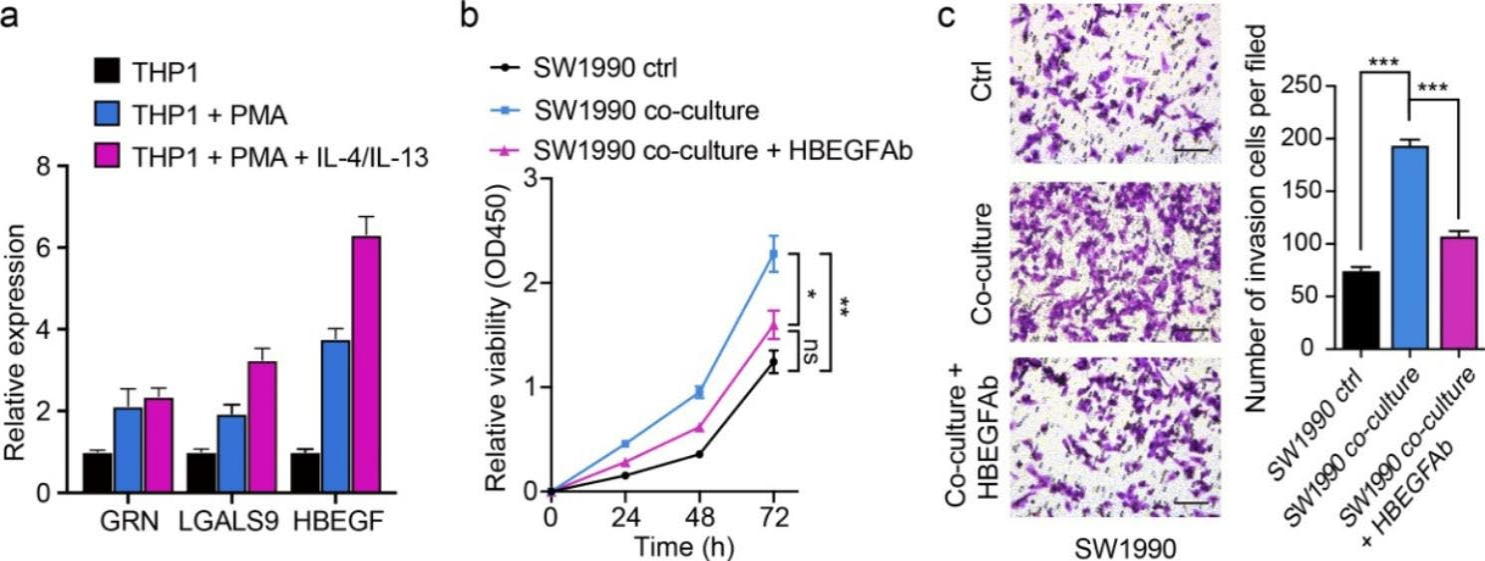
**TAMs-derived HBEGF promotes proliferation and invasion of SW1990. a** qRT-PCR evaluation of relative expressions of *GRN*, *LGALS9*, and *HBEGF* across three groups. **b** Line charts showing the levels of cell viability across the three groups (SW1990 alone, co-cultured SW1990, and co-cultured SW1990 with HBEGF blocking). The absorbance value at a wavelength of 450 nm was used as an indicator of cell viability. Data are presented as means ± SEM and analyzed with Student t-test (ns: non-significance; *P < 0.05; **P < 0.01). **c** Representative phase-contrast images of crystal violet stained SW1990 cells that invaded through the Matrigel matrix after 24 h of preprocessing (magnification 40×) across the three groups (SW1990 alone, co-cultured SW1990, and co-cultured SW1990 with HBEGF blocking). The quantitative analysis of invaded cells was performed by collecting five fields of view, and the data was presented as means ± SEM ( ***P < 0.001)

To see if *LGALS9*, *HBEGF*, and *GRN* mediate tumor-macrophage interactions and contribute to PDAC progression, we performed in vitro experimental studies for validation. By polarizing THP-1 cells into TAMs accordingly, *HBEGF* was confirmed to be overexpressed in the M2 polarized macrophages compared with uncompleted polarized status (Fig. 6a) and thus selected for further functional experiments. Then, the CCK-8 assay and Matrigel-coated Transwell system were used to observe cell growth and invasion of pancreatic cancer cells (SW1990 cell line). From the assays, we noted that growth and invasion rates were upregulated upon co-cultivation with TAMs compared with the cultivation of SW1990 alone (Fig. 6b-c). The effect was reversed with significance upon blockage with HBEGF-Ab (Blocking antibody) (Fig. 6b-c), validating the tumor-promoting role of TAMs-derived HBEGF.

## Discussion

Despite the progress in treatment strategies, the clinical outcomes of PDAC remain poor due to low resection rate and high relapse rate. Given the lacking of effective treatment strategies for PDAC, researchers have fostered ongoing efforts aiming at TME as targeted therapy. While significant heterogeneity of TME components, especially tumor-infiltrating myeloid cells, impedes our understanding of PDAC biology and hampers effective management strategy [34]. Herein, we provide a comprehensive single-cell transcriptomic atlas to characterize myeloid cells in PDACs. Our analysis reveals five macrophage subsets associated with diverse phenotypes and functions. Transcriptomic profiling and differentiation trajectory analysis demonstrated there is significant macrophage heterogeneity in the TME on continuums of inflammatory monocytes and RTMs to TAMs via proliferation macrophages. From the results of *CellphoneDB*, we observed that malignant ductal cells showed compromised crosstalk with inflammatory monocytes while maintaining close contact with cluster 1 and 3 macrophages. These two subsets were enriched with cytokine signatures and in the rapid proliferation stage, respectively, resembling the characteristics of TAMs [35]. TAMs constitute an essential compartment of the cancer-immune microenvironment and lead to an inherently immunosuppressed TME [36, 37]. Our result reveals the existence of active crosstalk between malignant cells and specific subsets of TAMs, which provide critical insights into TAM-oriented personalized therapeutic intervention.

Accumulating evidence suggests the importance of M1 and M2 TAMs in mediating PDAC progression [19, 38]. However, we could not clearly distinguish the M1 or M2 macrophage subset from the scRNA-seq dataset. Consistent with previous reports, there was almost no difference between M1 and M2 gene expression across different macrophage subsets [39, 40]. Instead, another classification system has been recently proposed that is more akin to their in vivo state whereby TAMs are subdivided into monocyte-like resident, alternatively activated TAMs [41]. It is hypothesized that M1 or M2 polarized state is more likely a concept indicating a functional property of pro-inflammation or anti-inflammation in particular settings rather than a specific cell compartment. M1-TAMs stand out to drive acinar-to-ductal metaplasia in the early phase of a neoplastic process. Also, it participates in cancer invasion by degradation of extracellular matrix [42, 43]. By contrast, in other settings like proliferation or vascularization, the M2-TAMs seize advantage [44]. M1 and M2 are nice concepts but are unfortunately the extremes of a continuum of intermediate cells. Instead, we hold the opinion that categorizing macrophages based on functional phenotype would be more reasonable.

Beyond characterizing the cellular composition of PDACs, it is crucial to understand how the diverse cellular components interact with each other to mediate PDAC progression. Presently, numerous studies have utilized scRNA-seq data to characterize cell-cell interaction [45, 46]. However, inter-tumoral heterogeneity tends to skew the results, limited to the small sample size. Here, we leveraged the advantages of bulk and single-cell transcriptome profiling to build a cell-cell interaction network, which identified myeloid and tumor cell lineages as interaction hubs among the TME. Further, profiling tumor-macrophage interactions identified several core ligand-receptor pairs, for instance, *CSF1-CSF1R* and *SPP1-CD44* axis, that coordinate tumor progression in mutual supporting ways [47, 48]. Moreover, accumulating evidence suggests the importance of these interaction signals in immunomodulation [49, 50].

Of note, our data demonstrated that Galectin-9-CD44/MET generates an autocrine loop with a unique value in predicting the prognosis of patients. Recent data highlighted the critical role of the Galectin-9-CD44 axis in regulating immune response [51]. Other studies have linked Galectin-9 to tolerogenic macrophage programming and adaptive immune suppression [52, 53]. As a novel immune checkpoint, Galectin-9 was reported to be available in periphery blood and demonstrated great value in disease diagnosing [54, 55]. Another Ligand-Receptor pair for concern in prognostic determination was granulin (*GRN*)-*EGFR*. It has been well-established that granulin facilitates pancreatic cancer metastasis and resistance to anti-PD-1 therapy [56, 57]. We uncovered an important role of HBEGF in the proliferation and invasion of SW1990 using in vitro experiments, consistent with a previous paper that macrophage-derived HBEGF was associated with a malignant course of PDAC [58], which might explain the limited efficacy of conventional therapy and poor prognosis. We concluded that immunotherapy targeting HBEGF markers might be effective for PDAC.

Our computational analysis serves as a rich resource of cellular states, function programs, and lineage ontology in macrophage compartments of PDAC. Our prediction of multiple intercellular interaction features between macrophage and malignant cells is implicated in coordinating cancer biology. Regarding limited immunotherapy options at hand, the prognostic tumor-macrophage interaction features represent potential targets for cancer control and aid in implementing immunotherapy approaches for PDAC.

## Electronic supplementary material

Below is the link to the electronic supplementary material.

Supplementary Material 1

Supplementary Material 2

## Data Availability

The single-cell dataset has been deposited to the Genome Sequence Archive with the accession number of PRJCA001063 by the original authors. All data and code in our study are available upon request by contacting Dr. Yang (lampirl@163.com).

## List of abbreviations

DCs: Dendritic cells
DEGs: Differentially expressed genes
ECM: Extracellular matrix
L-R: Ligand-Receptor
ORA: Over-representation
PCs: Principal components
PDAC: Pancreatic ductal adenocarcinoma
scRNA-seq: Single-cell RNA sequencing
ssGSEA: Single-sample gene set enrichment analysis
SCENIC: Single-cell regulatory network inference and clustering
RTMs: Resident tissue macrophages
RNA-seq: RNA sequencing
TAMs: Tumor-associated macrophages
TCGA: The Cancer Genome Atlas
TME: Tumor microenvironment
UMAP: Uniform manifold approximation and projection
