# Supplementary material for: Integrated transcriptional analysis reveals macrophage heterogeneity and macrophage-tumor cell interactions in the progression of pancreatic ductal adenocarcinoma
Source: BMC Cancer. 2023 Mar 2;23:199. doi: 10.1186/s12885-023-10675-y (PMC9983236; doi:10.1186/s12885-023-10675-y)
Supplement: Supplementary file 2 — Supplementary Material 2 [file 12885_2023_10675_MOESM2_ESM.doc]

**Supplymentary Figures**

**
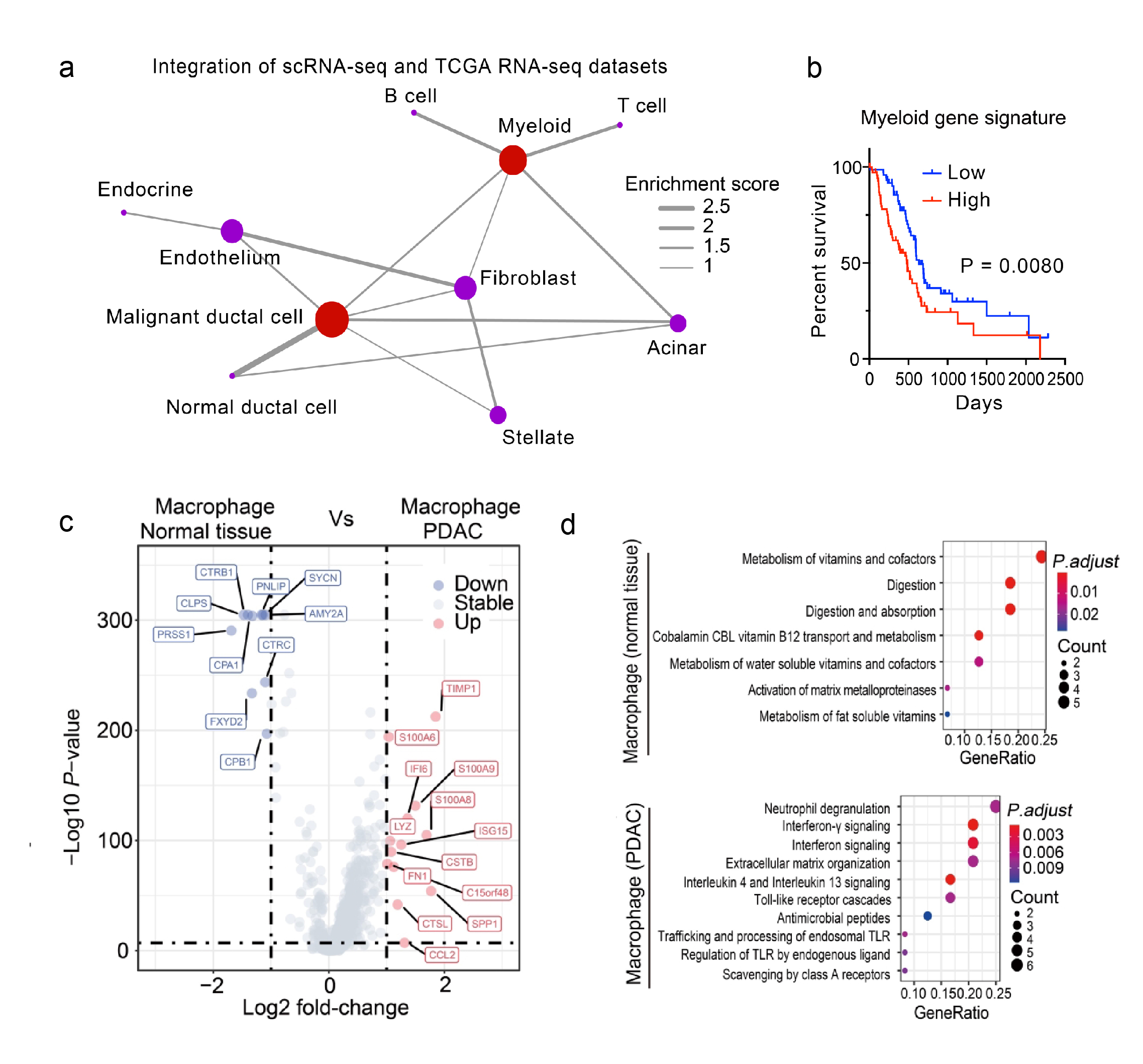
**

**Supplementary Fig. 1** **Identifying myeloid cells as an interactive hub of TME essential for PDAC progression.**

**a** Predicted correlative cell-cell interaction network of PDAC TME components based on scRNA-seq and TCGA bulk RNA-seq datasets. Nodes presented in the network indicate each TME component. Circle size indicates the interaction number of one cell type with others. Edges thickness indicates the enrichment score for each interaction. **b** Kaplan-Meier curves of the overall survival in different groups of PDAC patients stratified by signature scores of myeloid cells. P < 0.05 was identified as significant. **c** Volcano plot showing the DEGs between macrophages of normal and tumor tissue. Genes significantly upregulated or downregulated (log2 fold-change > 1, *P*-adjust value < 0.01) were marked with red or blue tags. **d** Dot plots showing the enrichment results for DEGs between macrophages of normal and tumor tissue. The index in the x-axis is the gene ratio of each term as defined through the *ClusterProfiler* R package. The bubble size indicates the number of genes in each term, and various colors correspond to different adjusted *P-*values, which are adjusted by the *Benjamini-Hochberg* method. PDAC, pancreatic ductal adenocarcinoma; DGEs, differential expressed genes.

**
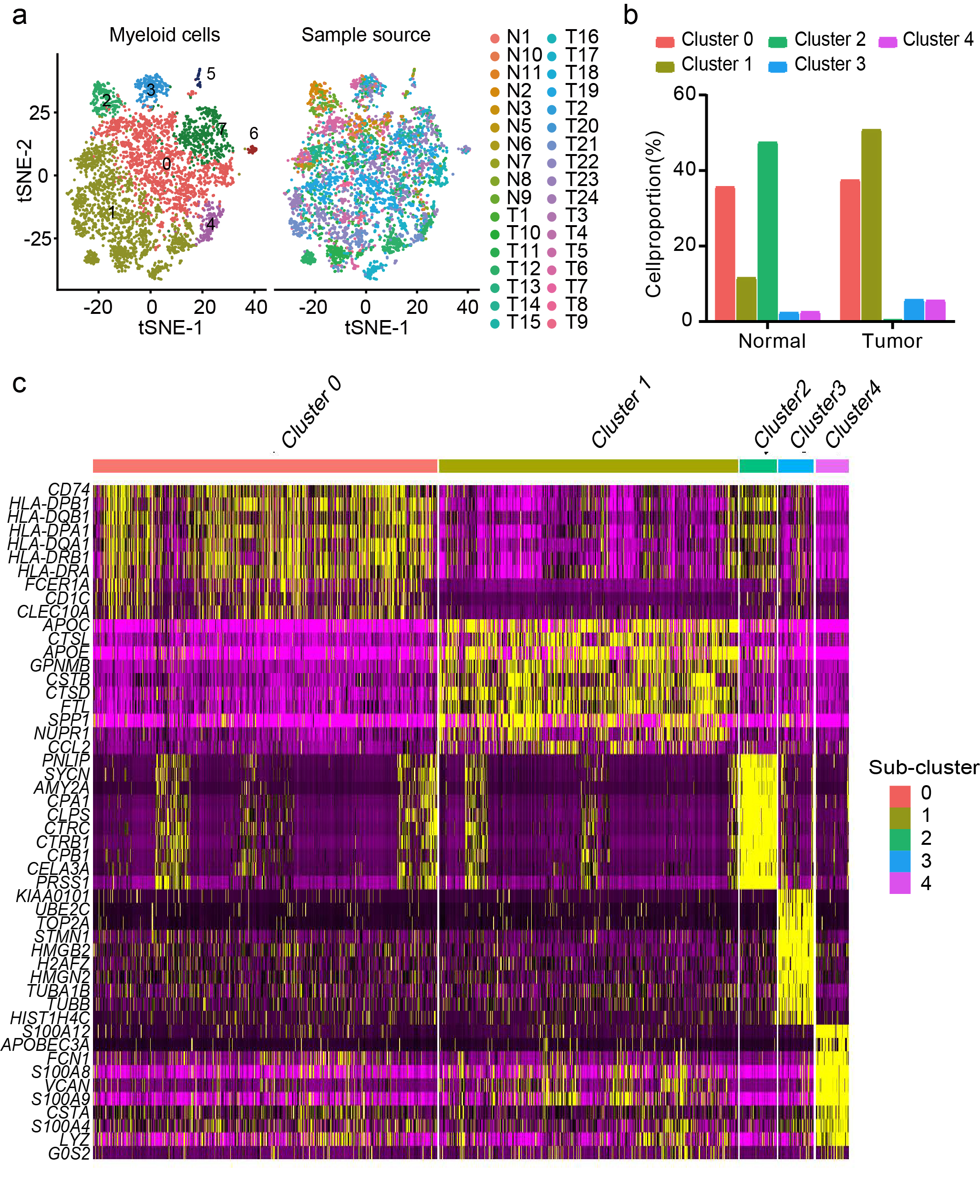
**

**Supplementary Fig. 2** **Sitewise cell proportions and top** **gene signatures across different** **macrophage subsets.**

**a** t-SNE projection of 5408 myeloid cells from PDAC tumors and normal tissues, colored according to graph-based clusterings (left panel) or patient source (right panel). **b** Bar plots showing the percentage of macrophage subsets across different sample sites. **c** Heatmap illustrating expression levels of top 10 gene signatures (log2 fold-change > 0.5, min.pct > 0.1) across different macrophage subsets.


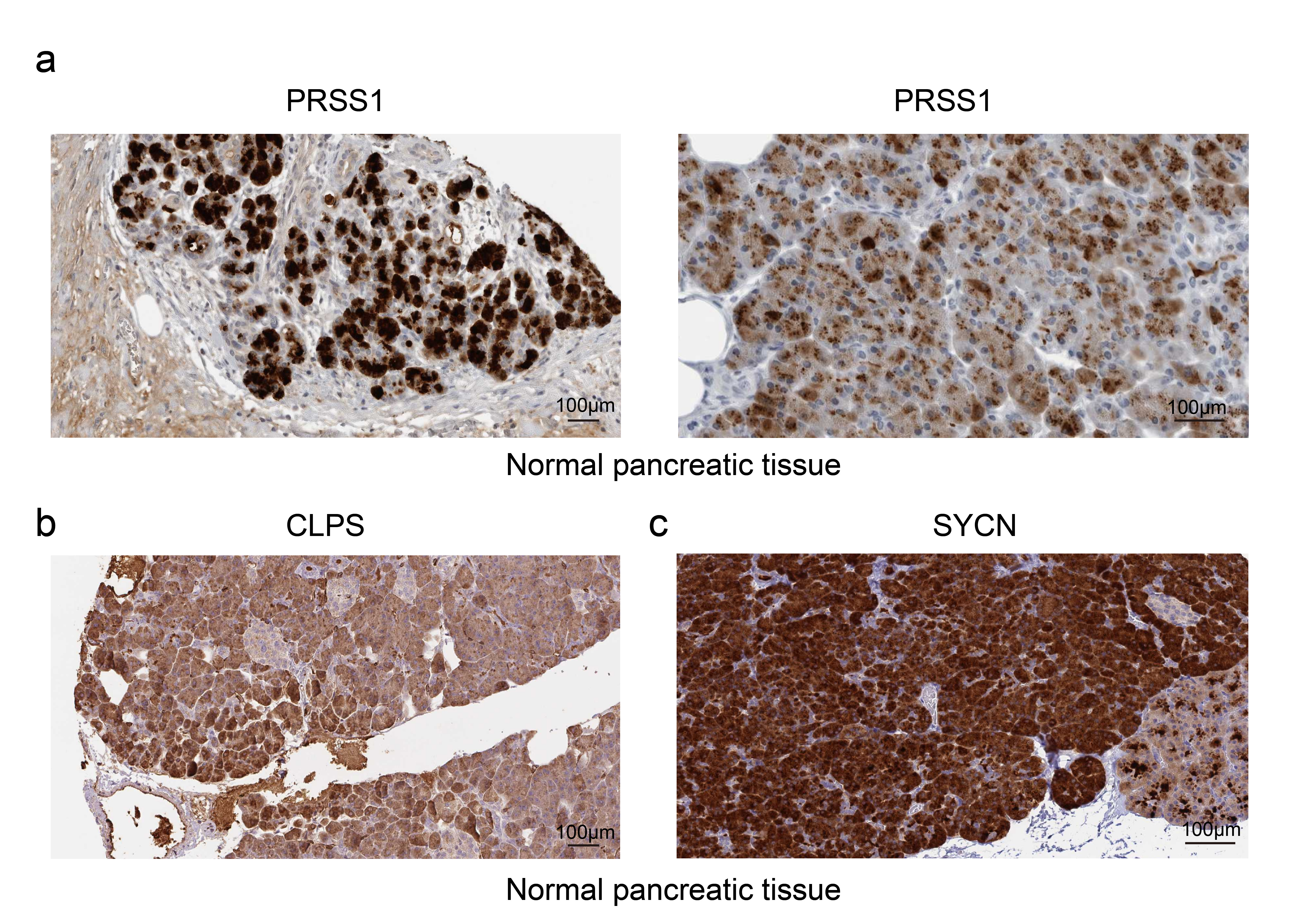


**Supplementary Fig. 3** **Immunohistochemical validation of tissue-resident macrophages markers in the normal pancreatic tissue.**

**a-c** Immunohistochemical data deposited in the Human Protein Atlas dataset showing the protein expression level of PRSS **(a)**, CLPS **(b)**, and SYCN **(c)** in the normal pancreatic tissue. Scale bar: 100μm.


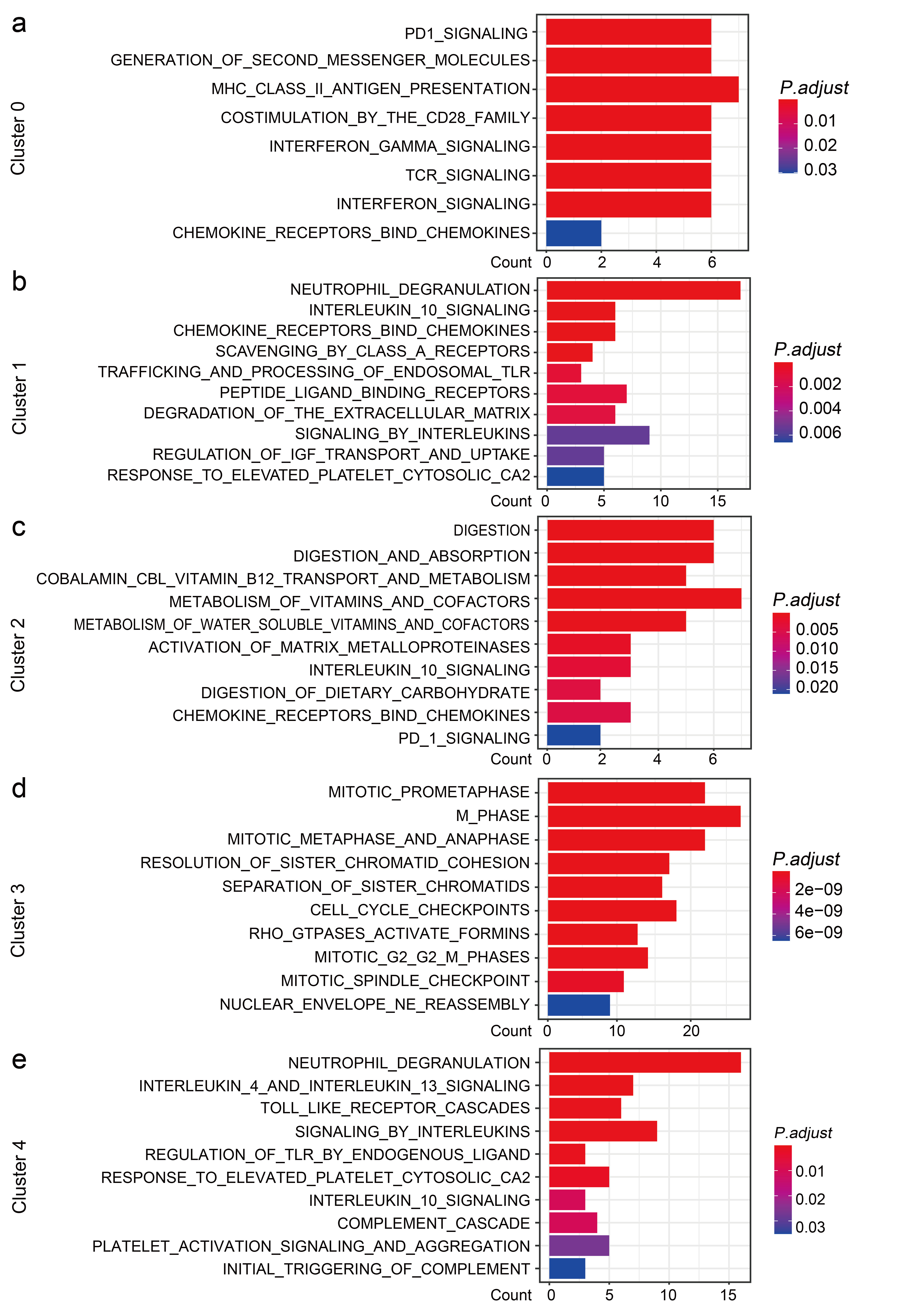


**Supplementary Fig. 4 Identification of** **functional pathways and modules for each** **macrophage subset.**

**(a-e)** Bar plots showing enrichment results of DEGs (min.pct>0.1, log2 fold-change>0.5) in each cluster versus other clusters using ORA (over-representation analysis). The index in the x-axis indicates the number of genes in each term and different colors correspond to different adjusted *P*-values.


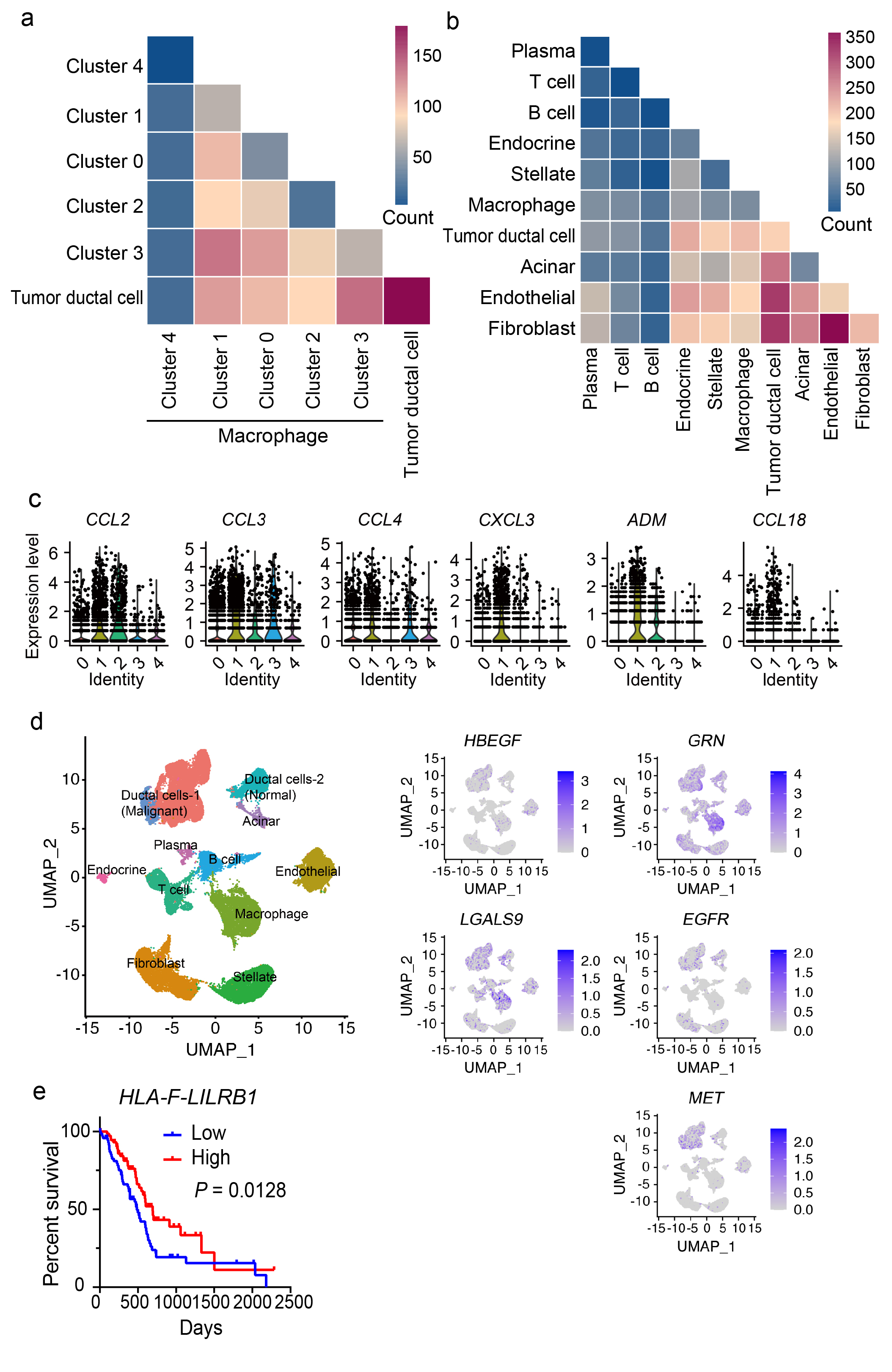


**Supplementary Fig. 5** **Inference of tumor-macrophage interactions. Data related to Fig. 5.**

**a** Heatmap showing the total number of ligand-receptor pairs between each macrophage subset and tumor cells. **b** Heatmap showing the total number of ligand-receptor pairs across tumor microenvironment components. **c** Violin plots present log-transformed, normalized expression levels of cytokines across the different clusters of macrophages. **d** Expression plots based on UMAP clustering depicted on PDAC datasets (left bottom panel). Normalized expression levels of the *HBEGF, LGALS9, GRN, EGFR,* and *MET* were projected onto the UMAP clustering (right panel). UMAP, uniform manifold approximation and projection. **e.** Kaplan-Meier curves of overall survival in different groups of PDAC patients stratified by the median expression level of the *HLA-F* and *LILRB1*.
